# Function, coping and health as predictors of sick leave after rehabilitation

**DOI:** 10.1093/eurpub/ckac131.287

**Published:** 2022-10-25

**Authors:** AM Berget, VP Moen, M Hustoft, J Assmus, LI Strand, JS Skouen, Ø Hetlevik

**Affiliations:** 1 Centre of Habilitation and Rehabilitation, Haukeland University Hospital, Bergen, Norway; 2 Department of Global Public Health and Primary Care, University of Bergen, Bergen, Norway; 3 Department of Health and Functioning, Western Norway University of Applied Sciences, Bergen, Norway; 4 Centre for Clinical Research, Haukeland University Hospital, Bergen, Norway; 5 Department of Physical Medicine and Rehabilitation, Haukeland University Hospital, Bergen, Norway

## Abstract

**Background:**

Function, coping and health are central factors in rehabilitation after injury or sickness. To investigate how these factors are associated with sick leave during 12 months after rehabilitation is the aim of this study.

**Methods:**

A sample of 412 rehabilitation patients ≤ 67years were included. They were all employed, and referred to inter-professional rehabilitation in western Norway. Rehabilitation consisted of physical activity/exercise, cognitive approaches and pain management. In two surveys patients reported mental (MCS) and physical (PCS) function (SF-36), self-perceived health (EQ-VAS) and coping (SOC-13). Register data on sick leave during 12 months in the calendar year after rehabilitation was retrieved from Statistics Norway and categorised to; non, (n = 168), ≤ 364 days (n = 152) and 365 days (n = 92). Ordinal regression was used to analyse association between sick leave and MCS, PCS, EQ-VAS and SOC-13, adjusted for age, sex and diagnoses.

**Results:**

The levels of MCS and PCS (SF-36) were found to be associated with sick leave; OR 0.96, 95% CI 0.92-0.99, p = 0.004 and OR 0.93, 95% CI 0.90-0.97, p < 0.001, respectively (Pseudo R2 = 0.1133). EQ-VAS and SOC-13 were significant predictors of sick leave in crude analysis (EQ-VAS: OR 0.97, 95% CI 0.96-0.98, p < 0.001. SOC-13: OR 0.98, 95% CI 0.95-0.98, p < 0.001), but not in the fully adjusted model (EQ-VAS: OR 0.98, 95% CI 0.96-1.01, p = 0.178. SOC-13: OR 0.99, 95% CI 0.99-1.03, p = 0.479).

**Conclusions:**

Patientś self-reported mental and physical function were associated with sick leave 12 months after inter-professional rehabilitation. Higher level of function was associated with no sick leave. In our study, patient’s self-reported health and coping were not associated with sick leave. This suggest that interventions for functional improvement are beneficial in health care strategies to help patients return to work after injury or sickness.

**Key messages:**

• Achieved higher physical and mental function after rehabilitation seems to contribute to reduced sick leave after injury or sickness.

• Improving function should remain a central factor in rehabilitation.

